# Sex-specific associations of matrix metalloproteinases in Alzheimer’s disease

**DOI:** 10.1186/s13293-023-00514-x

**Published:** 2023-05-23

**Authors:** Mari Aksnes, Trine H. Edwin, Ingvild Saltvedt, Rannveig S. Eldholm, Farrukh A. Chaudhry, Nathalie B. Halaas, Marius Myrstad, Leiv O. Watne, Anne-Brita Knapskog

**Affiliations:** 1 Department of Geriatric Medicine, University of Oslo, 0315 Oslo, Norway; 2 Department of Geriatric Medicine, Oslo University Hospital, 0450 Oslo, Norway; 3 Department of Neuromedicine and Movement Science, Norwegian University of Science and Technology, 7030 Trondheim, Norway; 4 Department of Geriatric Medicine, Clinic of Medicine, St. Olavs Hospital, Trondheim University Hospital, 7030 Trondheim, Norway; 5 Department of Molecular Medicine, University of Oslo, 0315 Oslo, Norway; 6 Department of Internal Medicine, Bærum Hospital, Vestre Viken Hospital Trust, 1346 Gjettum, Norway; 7 Department of Medical Research, Bærum Hospital, Vestre Viken Hospital Trust, 1346 Gjettum, Norway; 8 Institute of Clinical Medicine, Campus Ahus, University of Oslo, Oslo, Norway; 9 Department of Geriatric Medicine, Akershus University Hospital, Lørenskog, Norway

**Keywords:** Alzheimer disease, Biomarkers, Cerebrospinal fluid, Cognitive decline, Matrix metalloproteinases, Neurodegenerative diseases, Prognosis, Sex differences, Tissue inhibitor of metalloproteinases

## Abstract

**Introduction:**

Alzheimer’s disease (AD) can be characterised in vivo by biomarkers reflecting amyloid-β (Aβ) and tau pathology. However, there is a need for biomarkers reflecting additional pathological pathways. Matrix metalloproteinases (MMPs) have recently been highlighted as candidate biomarkers for sex-specific mechanisms and progression in AD.

**Methods:**

In this cross-sectional study, we investigated nine MMPs and four tissue inhibitors of metalloproteinases (TIMPs) in the cerebrospinal fluid of 256 memory clinic patients with mild cognitive impairment or dementia due to AD and 100 cognitively unimpaired age-matched controls. We studied group differences in MMP/TIMP levels and examined the associations with established markers of Aβ and tau pathology as well as disease progression. Further, we studied sex-specific interactions.

**Results:**

MMP-10 and TIMP-2 levels differed significantly between the memory clinic patients and the cognitively unimpaired controls. Furthermore, MMP- and TIMP-levels were generally strongly associated with tau biomarkers, whereas only MMP-3 and TIMP-4 were associated with Aβ biomarkers; these associations were sex-specific. In terms of progression, we found a trend towards higher MMP-10 at baseline predicting more cognitive and functional decline over time exclusively in women.

**Conclusion:**

Our results support the use of MMPs/TIMPs as markers of sex differences and progression in AD. Our findings show sex-specific effects of MMP-3 and TIMP-4 on amyloid pathology. Further, this study highlights that the sex-specific effects of MMP-10 on cognitive and functional decline should be studied further if MMP-10 is to be used as a prognostic biomarker for AD.

**Supplementary Information:**

The online version contains supplementary material available at 10.1186/s13293-023-00514-x.

## Abstract (plain language summary)

Analysis of the cerebrospinal fluid can provide insights into neurological diseases. In Alzheimer’s disease, the most common cause of dementia, low levels of the protein amyloid-β and high levels of phosphorylated tau in the cerebrospinal fluid are important biomarkers for diagnosis. However, analysis of other proteins might help us understand the cause of sex differences in Alzheimer’s disease. In this study, we have investigated a family of proteins called matrix metalloproteinases (MMPs) and tissue inhibitors of metalloproteinases (TIMPs). We have compared the levels of these proteins in patients with Alzheimer’s disease and age-matched controls without Alzheimer’s disease. We have investigated whether these proteins are associated with the established biomarkers amyloid-β and phosphorylated tau or disease progression; further, we studied whether there are differences between men and women. Two of the studied proteins, MMP-10 and TIMP-2, differed between the patients with Alzheimer’s disease and the controls. Generally, MMP- and TIMP-levels were strongly associated with phosphorylated tau, but only two markers were associated with amyloid-β: MMP-3 in men and TIMP-4 in women. In women, our results also suggest that higher MMP-10 could predict more cognitive and functional decline over time, but this finding is uncertain. Overall, our study supports the use of MMPs and TIMPs as biomarkers for sex differences and progression in Alzheimer’s disease.

**Supplementary Information:**

The online version contains supplementary material available at 10.1186/s13293-023-00514-x.

## Highlights

- MMPs and TIMPs can be useful markers of sex differences and disease progression in Alzheimer’s disease
- MMP-3 was associated with amyloid pathology in men but not in women and TIMP-4 was associated with amyloid pathology in women but not men
- We found a trend towards a female-specific effect of MMP-10 on cognitive and functional decline in Alzheimer’s disease patients.

**Supplementary Information:**

The online version contains supplementary material available at 10.1186/s13293-023-00514-x.

## Background

Alzheimer’s disease is a progressive neurodegenerative disorder characterised by amyloid-β (Aβ) and tau pathology. Biomarkers reflecting these pathological hallmarks can be measured in the cerebrospinal fluid (CSF) years to decades before symptom onset [1]. Beyond these established biomarkers, there is great interest in biomarkers reflecting additional pathological pathways such as neuroinflammation, which could be used as prognostic biomarkers or indicate mechanisms underlying sex differences in AD. Candidate biomarkers include matrix metalloproteinases (MMPs), a family of proteins with important roles in the modulation of neuroinflammation.

MMPs are proteases with several different substrates and are implicated in diverse physiological processes; for an overview of the 24 proteinases in the human MMP family, see for example Rivera et al. [2] Of especial relevance to AD, several MMPs have important roles in blood–brain barrier (BBB) permeability and neuroinflammation [3–7]. Moreover, several MMPs have direct interactions with Aβ and tau, and have higher expression in AD brains [8–10]. Indeed, MMPs are involved in Aβ degradation [11, 12], and MMPs have been found expressed in Aβ plaques [13, 14]. MMP-2, MMP-3 and MMP-9 are tau degrading enzymes [15], and in the AD brain active MMP-2 and MMP-9 colocalise with phosphorylated tau in neurofibrillary tangles [16, 17]. MMP activity is closely regulated by four tissue inhibitors of metalloproteinases (TIMPs) [7]; TIMP-1, TIMP-2, TIMP-3 and TIMP-4. These TIMPs have also been linked to AD pathology and are upregulated in early disease stages in AD models [18–21].

Several MMPs and TIMPs have been studied in the CSF as potential biomarkers for AD, with mixed results [22–30]. However, MMPs have only recently been highlighted as promising candidate biomarkers for studying progression in AD [31]. Indeed, selected MMPs (MMP-2 and MMP-10) have recently been linked to conversion from mild cognitive impairment (MCI) to AD dementia [31, 32]. These proteins could be prognostic biomarkers that are independent of Aβ and tau pathology [31], but their impact on cognitive and functional decline in patients with established cognitive impairment remain to be explored. Simultaneously, MMPs have been highlighted as candidate biomarkers for sex-specific mechanisms driving neurological disorders such as AD [33], in part due to their interactions with the hormone 17β-oestradiol [34]. In line with this, sex differences in MMP-3 levels and sex-specific interactions with cognitive function have been reported [35, 36]. Nonetheless, few studies have aggregated data by sex [33].

The purpose of the current study was to investigate nine MMPs and four TIMPs in a memory clinic cohort with AD and elaborate on the associations with established markers of Aβ and tau pathology as well as functional and cognitive decline over time. Moreover, we aimed to investigate sex-specific interactions.

## Methods

### Study design

This was a cross-sectional study including patients from two Norwegian memory clinics (128 patients from Oslo University Hospital and 128 patients from St. Olav University Hospital, Trondheim) included in the Norwegian Registry of Persons Assessed for Cognitive Symptoms (NorCog) and 100 cognitively unimpaired (CU) controls recruited to the Cognorm study from surgical departments at Oslo University Hospital and Diakonhjemmet Hospital (Oslo, Norway).

### Memory clinic patients

Patients were included in NorCog between 2009 and 2018. Patients diagnosed with MCI-AD or AD dementia who had undergone lumbar puncture and had biomarker evidence of Aβ pathology (i.e. were A+, see “AT(N)-classification”﻿) were included (*n* = 256).

Clinical diagnoses using criteria for research were made upon inclusion in the study by reviewing the patient journals. Results of the *APOE* genotyping were not available at the time of diagnosis. The clinical diagnoses of probable or possible dementia or MCI-AD were made using the National Institute of Health and the Alzheimer’s Association 2011 criteria [37, 38]. Patients with concomitant aetiologically mixed AD and cerebrovascular disease were included, but patients with other mixed presentations were excluded. The two memory clinics in the present study arrange weekly diagnostic consensus meetings to harmonise diagnoses, wherein the researchers also participate.

Patients were assessed according to a standardised research protocol by experienced memory clinic physicians [39]. The assessment comprised a battery of several cognitive tests including the mini-mental status examination (MMSE), the Consortium to Establish a Registry of Alzheimer’s Disease 10-item word list and figure copying tests, the clock drawing test (CDT), and the trail making tests A and B. Moreover, the patients underwent a thorough physical examination including MRI brain scans, blood sampling and lumbar puncture. The AD CSF core biomarkers, i.e. Aβ_42_, phosphorylated tau threonine 181 (p-tau) and total tau (t-tau), were analysed at the Department of Interdisciplinary Laboratory Medicine and Medical Biochemistry at Akershus University Hospital (Lørenskog, Norway) using the Innotest kit enzyme-linked immunosorbent assays (Innogenetics, Ghent, Belgium). *APOE* genotyping was performed at deCODE Genetics (Reykjavik, Iceland) using the Illumina Infinium OmniExpress v1.1 chip.

### Cognitively unimpaired controls

The CU controls were aged 65 or older and had been referred for elective surgery in spinal anaesthesia due to gynaecological, orthopaedic or urological problems in 2012–2013; CSF was collected prior to the spinal anaesthesia procedure. All patients with available CSF for analysis were candidates for inclusion (*n* = 140). The CU controls completed the same cognitive test battery as the patients, and only individuals with normal test results (according to age and education adjusted norms) at baseline and available AD biomarkers were included in the current study (*n* = 100). For further information about the CU group, see Idland and colleagues [40]. The AD core CSF biomarkers were analysed at Sahlgrenska University Hospital (Mölndal, Sweden) using the Innotest kit enzyme-linked immunosorbent assays (Innogenetics, Ghent, Belgium). *APOE* genotyping was performed at Applied Biosystems (Carlsbad, CA, USA) using TaqMan Allelic Discrimination technology.

### AT(N)-classification

In line with the 2018 National Institute of Health and the Alzheimer’s Association research framework [1], the cohort was AT(N)-classified based on established CSF biomarkers reflecting Aβ pathology (A), tau pathology (T) or neurodegeneration (N) [1]. Specifically, low CSF Aβ_42_ was classified as A+, high CSF p-tau as T+ and high CSF t-tau as N+. Whilst MRI examinations were available for most of the cohort, these scans have been performed in different locations and using different protocols, and therefore N-classification based on MRI data was not performed. Whilst CSF from both cohorts was analysed using Innotest kits, laboratory recommended cut-offs were applied due to established variabilities between different laboratories [41]. For the memory clinic patients, cut-offs for a normal test were Aβ_42_ > 700 pg/mL; p-tau < 80 pg/mL; and t-tau < 300 pg/mL for patients under 50 years, < 450 pg/mL for patients aged 50–70 years and < 500 pg/mL for patients older than 70 years. For the CU controls, cut-offs for a normal test were Aβ_42_ > 530 pg/mL, p-tau < 60 pg/mL and t-tau < 350 pg/mL.

### Measurement of MMPs and TIMPs

All CSF samples were analysed by Eve Technologies (Calgary, Canada) using a 13-plex Discovery Assay^®^ on a Luminex^®^ xMAP^®^ instrument. This assay simultaneously measures 13 MMP and TIMPs, MMP-1, MMP-2, MMP-3, MMP-5, MMP-8, MMP-9, MMP-10, MMP-12, MMP-13, TIMP-1, TIMP-2, TIMP-3, TIMP-4, in a single microwell. All samples were measured in duplicate. More details on the measurement of the MMPs and TIMPs are provided in Additional file 1: Table S1.

### Clinical progression

For the patient cohort, the extent of cognitive and functional impairment was scored post hoc by certified raters using the clinical dementia rating (CDR) scale. The categories memory; orientation; judgment and problem‐solving; community affairs, home and hobbies; and personal care were given a score of either 0, 0.5, 1, 2, or 3, with higher score signifying more severe impairment [42]. The summed score from all categories, the CDR sum of boxes (CDR-SB) [43], was used in the analyses. The clinical evaluation closest to lumbar puncture was considered the baseline; the average time between baseline and lumbar puncture was 62 days (standard deviation = 45 days). Follow-up was restricted to 3 years to limit survival bias.

### Statistical analysis

Statistical analyses were performed in STATA 16.1 and data visualisations were created in R4.1.1 using RStudio. Categorical variables were compared using the χ^2^ test. Continuous variables were compared across more than two groups using one-way ANOVAs. In cases of heterogeneity of variances across groups, the non-parametric Kruskal–Wallis test was used as an alternative. The Wald test was used for post hoc comparisons after ANOVA and the Dunn tests was used for post hoc comparisons after Kruskal–Wallis. For post hoc tests, Holm’s method was used to adjust for multiple comparisons and adjusted *P-*values were reported. Continuous variables were compared across men and women using independent samples *t*-test. T-tau and p-tau were closely correlated in our cohort, and to avoid collinearity only p-tau was included as a covariate in the statistical analyses. *APOE* ε4-status was coded as carrier/non-carrier.

To investigate the effects of AD biomarkers on MMP/TIMP levels, we performed multiple linear regression with each MMP/TIMP as a dependent variable. Each regression model included both A-status and T-status as independent variables and was adjusted for age, sex, *APOE* ε4-status and stage of cognitive impairment (CU, MCI or dementia). To identify any sex-specific effects, sex-stratified multiple linear regressions were also conducted. To test whether baseline MMP/TIMP levels were associated with cognitive and functional decline in memory clinic patients, we performed linear mixed-effects models on the patients with at least one follow-up examination (*n* = 193). We performed one regression analysis for each MMP/TIMP with CDR-SB as the dependent variable. The fixed effects were the specified CSF MMP/TIMP × time, age, sex, baseline CSF Aβ_42_, baseline CSF p-tau and stage of cognitive impairment (MCI or dementia). To identify any sex-specific effects, sex-stratified linear mixed-effects regressions were also conducted. Only cases with complete data were included in the regression analyses.

## Results

### Group differences

The analysis included 256 patients (50 MCI-AD and 206 AD dementia, all A+) and 100 CU controls (74 A− and 26 A+). The clinical profiles of these four groups are presented in Table 1. There was a significantly higher proportion of women in the AD dementia group (58.7%) compared to the CU A− group (41.9%, χ^2^ = 6.23, *P* = 0.01). As expected, the A+ groups had significantly more *APOE* ε4 carriers (77.7%) than the CU A− group (28.6%, χ^2^ = 59.71, *P* < 0.01). Moreover, the patients performed worse on the MMSE and had a higher proportion of T+ and N+ individuals compared to the control groups.

**Table 1:** Clinical profile of the CU A−, CU A+, MCI-AD and AD dementia patients

	CU A−	CU A+	MCI-AD	AD dementia	χ^2^/F (df)	*P* (η^2^)
*N*	74	26	50	206		
Female *n* (%)	31 (41.9)	15 (57.7)	27 (54.0)	121 (58.7)	6.35	0.10
Age	72.2 (6.1)	72.2 (5.9)	71.5 (4.7)	70.0 (6.7)	2.64	0.45^d^
*APOE* ε4 *n* (%)^f^	20 (28.6)	16 (69.6)^a^	40 (85.1)^a^	139 (76.8)^a^	**61.63**	**< 0.01**
Education	14.8 (3.5)	12.4 (3.1)^a^	13.1 (3.8)	12.2 (3.7)^a^	8.95 (3)	**< 0.01**
MMSE	29.2 (1.0)	29.0 (0.8)	26.3 (3.3)^a, b^	22.2 (4.3)^a−c^	**192.1**	**< 0.01**^d^
CDT	4.8 (0.5)	4.7 (0.6)	4.1 (1.0)	3.3 (1.5)	33.45 (4)	0.94
CDR-SB	NA	NA	1.9 (1.4)	4.4 (2.0)^c^		**< 0.01**^e^
CSF Aβ_42_	806.7 (137.6)	443.2 (76.2)	537.9 (92.0)	512.4 (101.9)		NA
CSF p-tau	57.4 (17.7)	58.0 (22.2)	86.6 (36.2)	98.8 (43.4)		NA
T +	26 (35.1)	11 (42.3)	28 (56.0)^a^	138 (67.0)^a, b^	**25.14**	**< 0.01**
CSF t-tau	349.8 (123.2)	365.8 (170.9)	627 (284.6)	766.4 (378.6)		NA
N +	32 (43.3)	11 (42.3)	35 (70.0)^a, b^	158 (76.7)^a, b^	**34.58**	**< 0.01**

Data reported is mean (standard deviation) unless otherwise indicated. *P-*values are for comparisons between all four groups using χ^2^ (categorical variables) or ANOVA (continuous variables), unless otherwise specified. Significant differences in bold. Biomarkers are given in pg/mL. Comparison of biomarker levels between CU controls and patients is not applicable as different cut-offs were applied

^a^Significantly different from the CU A− group; ^b^Significantly different from the CU A + group; ^c^Significantly different form the MCI-AD group; ^d^Kruskal–Wallis test; ^e^independent samples *t*-test; ^f^*n* = 321, 35 missing genotype. *A+*: biomarkers positive for Aβ pathology, *Aβ* amyloid-β, *AD* Alzheimer’s disease, *ANOVA* analysis of variance, *APOE* apolipoprotein E, *CDR-SB* clinical dementia rating scale sum of boxes, *CDT* clock drawing test, *CSF* cerebrospinal fluid, *CU* cognitively unimpaired, *MCI* mild cognitive impairment, *MMSE* mini-mental status examination, *N +* biomarkers positive for neurodegeneration, *p-tau* phosphorylated tau, *T +* biomarkers positive for tau pathology, *t-tau* total tau

Overall, there was a significantly higher percentage of amyloid positive women (84.0%) than men (73.5%) in the cohort, χ^2^ = 5.98, *P* = 0.01. On average, the men had longer educations (mean = 13.5 years, standard deviation [SD] = 4.24) than the women (mean = 12.4, SD = 3.30, *P* = 0.01). Clinical profiles for the included men and women are presented in Additional file 1: Table S2.

### MMP- and TIMP-levels in Alzheimer’s disease

**Fig. 1:**
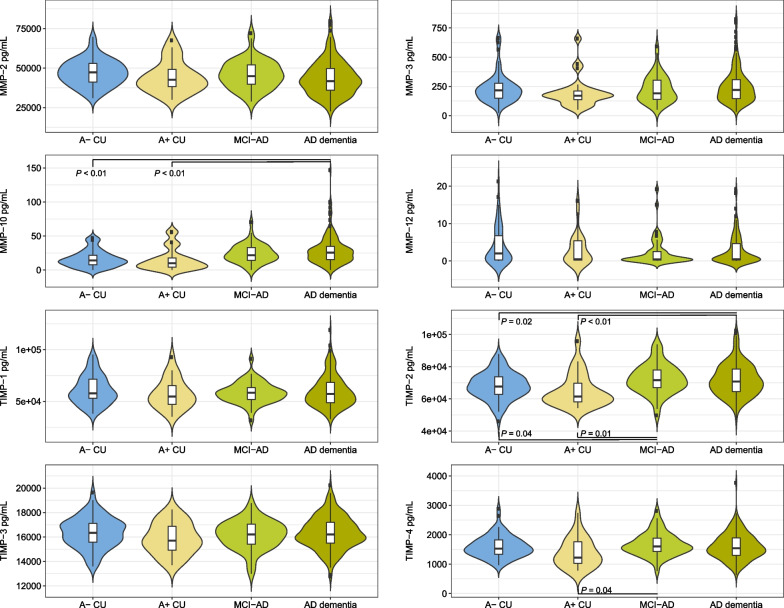
Violin plots showing the distribution of MMP- and TIMP-levels across the A− CU, A+ CU, MCI-AD and AD dementia groups. Boxes show the median (middle line), first quartile (bottom edge) and third quartile (top edge). *P-*values are given for Holm post hoc comparisons after ANOVA. *AD* Alzheimer’s disease, *CU* cognitively unimpaired, *MCI* mild cognitive impairment, *MMP* matrix metalloproteinase, *TIMP* tissue inhibitor of matrix metalloproteinase

MMP-1, MMP-7, MMP-8, MMP-9 and MMP-13 were detectable in less than 40% of samples and were therefore excluded from the analysis. In a minority of samples, the levels of MMP-10 (2.8%) and MMP-12 (37.2%) were below the lower limit of quantification; values below this limit were replaced with a random value between 0 and the lower limit of quantification. MMP-2, MMP-3 and all the TIMPs were detectable in all samples. The distributions of MMP- and TIMP-levels across the CU A−, the CU A +, the MCI-AD and AD dementia groups are presented in Fig. 1.

The average MMP- and TIMP-levels across the groups are presented in Table 2. There was an overall group difference for MMP-10 and TIMP-2 levels, *P* < 0.01. Post hoc comparisons revealed significantly higher MMP-10 levels in AD dementia patients (mean = 28.4 pg/mL) and MCI-AD patients (24.0 pg/mL) compared to CU A− (16.5 pg/mL, *P* < 0.01) and CU A+ controls (15.4 pg/mL, *P* < 0.01). For TIMP-2 levels, post hoc comparisons revealed significantly higher levels in MCI-AD patients (72.4 ng/mL) compared to CU A− (68.1 ng/mL, F = 5.99, degrees of freedom [df] = 1, *P* = 0.04) and CU A+ (65.2 ng/mL, F = 9.70, df = 1, *P* = 0.01) controls. TIMP-2 levels were also significantly higher in the AD dementia patients (71.8 ng/mL) compared to the A− and A+ CU controls (F = 8.04, df = 1, *P* = 0.02, and F = 10.97, df = 1, *P* < 0.01, respectively). While there was no overall group difference for TIMP-4 levels, post hoc comparisons revealed significantly lower TIMP-4 levels in the A + CU controls (1.4 ng/mL) compared to the MCI-AD patients (1.7 ng/mL, F = 7.47, df = 1, *P* = 0.04).

**Table 2:** CSF MMP/TIMP levels in CU A−, CU A+, MCI-AD and AD dementia patients

	CU A−	CU A+	MCI-AD	AD dementia	χ^2^/F (df)	*P* (η^2^)
MMP-2 (ng/mL)	47.1 (8.3)	44.2 (9.9)	45.9 (9.0)	43.6 (10.8)	2.45 (3)	0.06
MMP-3 (pg/mL)	232.6 (127.3)	197.8 (129.7)	227.7 (115.6)	250.1 (143.6)	2.43 (3)	0.23
MMP-10 (pg/mL)	16.5 (12.3)	15.4 (16.4)	24.0 (13.6)^a,b^	28.4 (20.1)^a, b^	38.2	**< 0.01**^**c**^
MMP-12 (pg/mL)	4.0 (4.9)	3.1 (4.1)	2.4 (3.8)	2.7 (3.6)	2.50	0.48^c^
TIMP-1 (ng/mL)	61.1 (13.8)	57.2 (14.1)	58.2 (10.2)	59.4 (15.0)	0.68 (3)	0.57
TIMP-2 (ng/mL)	68.1 (8.4)	65.2 (10.2)	72.4 (9.2)^a, b^	71.8 (9.9)^a, b^	**6.08 (3)**	**< 0.01 (0.05)**
TIMP-3 (ng/mL)	16.4 (1.2)	15.9 (1.3)	16.2 (1.2)	16.3 (1.3)	1.21 (3)	0.31
TIMP-4 (ng/mL)	1.6 (0.4)	1.4 (0.5)	1.7 (0.4)^b^	1.6 (0.4)	2.55 (3)	0.06

*P-*values are given for comparisons between all four groups using ANOVA, unless otherwise specified. Significant differences in bold

^a^Significantly different from the CU A− group; ^b^Significantly different from the CU A+ group; ^c^Kruskal–Wallis test. *A+*: biomarkers positive for Aβ pathology, *AD* Alzheimer’s disease, *ANOVA* analysis of variance, *CSF* cerebrospinal fluid, *CU* cognitively unimpaired, *MCI* mild cognitive impairment, *MMP* matrix metalloproteinase, *TIMP* tissue inhibitor of matrix metalloproteinase

On average, men had higher levels of all measured MMPs and TIMPs, except for MMP-12 and TIMP-4; see Additional file 1: Table S3.

### Effect of amyloid- and tau-biomarker-status

To investigate whether MMP/TIMP-levels were associated with A− or T-biomarker status, multivariate linear regressions were conducted with each of the measured MMPs and TIMPs as dependent variables; see Table 3. In addition to A− and T-status, age, sex, *APOE* ε4-status and stage of cognitive impairment (CU, MCI or dementia) were included as covariates. For MMP-12, a good model fit could not be achieved with multiple regression (adjusted R^2^ = 0.01); therefore MMP-12 levels were not further analysed.

**Table 3:** Adjusted effects of A− and T-status on MMP- and TIMP-levels in CU controls and patients

Dependent variable/independent variable	MMP-2	MMP-3	MMP-10	TIMP-1	TIMP-2	TIMP-3	TIMP-4
A+ status	− 0.13	− 0.18*	− 0.07	− 0.14	− 0.11	− 0.19	− 0.25**
T+ status	0.24**	0.39**	0.37**	0.16**	0.16**	0.17**	0.43**
Age	0.31**	0.11*	0.13**	0.36**	0.22**	0.11*	0.05
Male sex	0.32**	0.18**	0.20**	0.26**	0.34**	0.27**	0.04
*APOE* ε4 carrier	0.05	0.06	0.08	0.13*	0.05	0.10	0.14*
MCI (vs. CU)	0.07	0.09	0.16*	0.01	0.26**	0.07	0.16*
Dementia (vs. CU)	0.00	0.19	0.33**	0.10	0.35**	0.16	0.12
Adjusted R^2^	0.28	0.20	0.29	0.23	0.24	0.11	0.19

Standardised β-coefficients are presented. *P < 0.05, ** *P* < 0.01. *A+*: biomarkers positive for Aβ pathology, *APOE*: apolipoprotein E, *CU* cognitively unimpaired, *MCI* mild cognitive impairment, *MMP* matrix metalloproteinase, *T+*: biomarkers positive for tau pathology, *TIMP* tissue inhibitor of matrix metalloproteinase

Tau-positivity was significantly associated with increased levels of all measured MMPs and TIMPs. In contrast, amyloid-positivity was only significantly associated with reduced levels of MMP-3 and TIMP-4, and for these markers tau-positivity had a larger effect. As such, MMP and TIMP-levels appear to be more strongly associated with indicators of tau-pathology rather than amyloid pathology. In terms of the other covariates, increased age and male sex were significantly associated with increased levels of all markers except TIMP-4. The *APOE* ε4 genotype predicted significantly higher levels of TIMP-1 (β = 0.13, *P* = 0.02) and TIMP-4 (β = 0.14, *P* = 0.02). Compared to the CU reference group, cognitive impairment at the MCI stage was associated with increased levels of TIMP-2 and TIMP-4, whereas the dementia stage was associated with increased levels of MMP-10 and TIMP-2.

### Sex-specific effects

Sex-stratified analyses revealed that T+ patients in both sexes had increased levels of all markers compared to T− patients, except for TIMP-1 where a significant effect was only found for men (β = 0.22, *P* < 0.01). Age remained associated with higher MMP-2, TIMP-1 and TIMP-2 levels for both sexes, but the effect of age on MMP-3, MMP-10 and TIMP-4 levels was exclusive to women. The *APOE* ε4 genotype was not associated with any MMPs or TIMPs in women, but among the male patients *APOE* ε4 carriers had increased levels of MMP-10 (β = 0.22, *P* = 0.01), TIMP-1 (β = 0.28, *P* < 0.01), TIMP-2 (β = 0.18, *P* = 0.04) and TIMP-3 (β = 0.26, *P* < 0.01) compared to non-carriers. Amyloid-positivity was associated with decreased levels of MMP-3 levels in men (β = − 0.41, *P* = 0.01) and on TIMP-4 levels in women (β = − 0.24, *P* = 0.04) compared to amyloid-negativity. Finally, compared to the CU reference group MCI and dementia were associated with increased MMP-10 levels in women (MCI β = 0.22, *P* < 0.01 and dementia β = 0.37, *P* < 0.01). Compared to the CU stage, the dementia stage was associated with increased TIMP-2 levels for both sexes, but a significant effect of MCI was only evident in women (β = 0.32, *P* < 0.01).

### Disease progression

Follow-up data were available for 193 patients. Median follow-up time was 2 years (25th percentile = 1 year and 1 months, 75th percentile = 2 years and 4 months) and the median number of follow-up visits were 3 (25th percentile = 2, 75th percentile = 4). After adjusting for age, sex, baseline levels of Aβ_42_ and p-tau and clinical stage (dementia vs MCI), there were no significant interaction effects with time on CDR-SB scores for any MMPs or TIMPs.

When comparing MMP and TIMP levels in MCI patients who converted to dementia (*n* = 14) with MCI patients who did not convert to dementia during the follow-up period (*n* = 36), only TIMP-2 differed between the groups; TIMP-2 levels were higher in MCI patients who converted (77.6 ng/mL) compared to those who did not (70.3 ng/mL, *P* = 0.01).

### Sex-specific effects

Follow-up data were available for 110 women and 83 men. Median follow-up time was 2 years for women (25th percentile = 1 year and 2 months, 75th percentile = 2 years and 5 months) and 1 year and 9 months for men (25th percentile = 11 months, 75th percentile = 2 years and 3 months). The median number of follow-up visits were 3 (25th percentile = 2, 75th percentile = 4) for both women and men. In sex-stratified analyses, after adjusting for age, baseline levels of Aβ_42_ and p-tau and clinical stage (dementia versus MCI) there were no significant interactions effects of time × MMP/TIMP on CDR-SB scores. However, there was a trend towards a significant effect of time × MMP-10 on CDR-SB scores in women (β = 0.022, standard error = 0.012, *P* = 0.06), but not in men (β = − 0.002, standard error = 0.009, *P* = 0.84).

Sex-specific effects in MCI patients only were not assessed due to limited group sizes.

## Discussion

In the present study, we have demonstrated that MMP-10 and TIMP-2 levels differed significantly between cognitively impaired individuals compared to A+ and A− CU controls. Furthermore, we found that MMP and TIMP-levels were strongly associated with tau biomarkers, whereas only MMP-3 and TIMP-4 were associated with amyloid biomarkers; these associations with amyloid were sex-specific. In terms of progression, baseline MMP and TIMP levels did not predict faster cognitive and functional decline in our memory clinic cohort. However, we found a trend suggesting that higher MMP-10 at baseline may predict more cognitive and functional decline over time in women, but not in men.

Our finding of increased MMP-10 levels in AD patients compared to the CU controls is in line with previous research [24]. However, increased levels of MMP-10 have previously been shown in MCI-AD compared to CU controls [25, 26], which was not replicated here. While several previous studies have reported altered levels of MMP-2 and MMP-3 in AD [22, 23, 28, 44], we did not find any differences in these markers between AD patients and CU controls. In the current study, all measured MMPs and TIMPs were associated with tau-positivity. This is in line with previous research linking MMP-2, MMP-3 and MMP-10 to tau biomarkers [24, 28, 29]. Moreover, reduced levels of MMP-3 and TIMP-4 were associated with amyloid-positivity. In line with this, reduced levels of MMP-3 have been found in CSF with low levels of Aβ_42_, [29, 44] and an exploratory study on CU individuals over 60 years has suggested an Aβ-associated effect of MMP-3 on brain atrophy [32]. To our knowledge, TIMP-4 has not previously been linked to amyloid pathology, but increased plasma levels of TIMP-4 have been linked to AD risk and cognitive impairment [45]; the association between Aβ pathology and TIMP-4 merits further investigation.

There were several sex-specific interactions between MMPs/TIMPs and other covariates, including sex-specific effects of amyloid-positivity on MMP-3 for men and TIMP-4 for women. Sex differences in MMP-3 in AD have previously been reported in human plasma, human brains, and animal models [35, 36]. In line with our findings, MMP-3 levels are increased in the brain and correlate more closely with markers of AD neuropathology and cognitive impairment in men [36]. However, an association between MMP-3 and Aβ has also been reported in CU women. Interestingly, the same study demonstrated that MMP-3 degrades nerve growth factor and was associated with markers of nerve growth factor dysmetabolism in men. The authors speculate that MMP-3 could contribute to nerve growth factor dysmetabolism and consequent cholinergic atrophy in a sex-specific, possibly Aβ-dependent manner [36]. Our results suggest that sex-specific effects of MMP-3 on AD pathology should be investigated further together with TIMP-4. We also found a male-specific effect of *APOE* ε4 on MMP-10, TIMP-1, TIMP-2 and TIMP-3, and a female-specific effect of age on MMP-3, MMP-10, and TIMP-4. Notably, increased age and the *APOE* ε4 allele are central risk factors for AD, but as it is well-documented that *APOE* ε4 confers a higher risk for AD in especially in women, the male-specific effects of *APOE* ε4 warrant further investigation. [46–48].

In the memory clinic cohort, baseline MMP and TIMP levels did not predict faster functional and cognitive decline over time, although TIMP-2 levels were found to be increased in MCI patients who converted to dementia. Furthermore, sex-stratified analyses suggest that higher baseline MMP-10 levels might be associated with faster functional and cognitive decline in women, but not in men. This is in line with recent findings reporting that higher MMP-10 levels are associated with increased risk of conversion from MCI to AD dementia [31], and highlights that MMP-10 levels may also provide prognostic information for patients at the dementia stage. Increased CSF MMP-10 levels have also been linked to disease progression in Parkinson’s disease [49], suggesting a more general role for MMP-10 in the progression of neurodegenerative disorders. Indeed, it has been speculated that MMP-10 protein levels are markers of disease-independent pathways related to ageing [31]. Notably, in our cohort the association of MMP-10 levels with functional and cognitive decline was female-specific, and we also found a female-specific effect of age on MMP-10 levels. As it has recently been proposed to incorporate MMP-10 into the AT(N)-framework as a prognostic marker [31], our results highlight that sex-specific mechanisms linking ageing, MMP-10 and clinical progression must be investigated further.

One limitation of the current study is that the core biomarkers were analysed in laboratories with different recommended cut-offs, precluding the use of CSF Aβ_42_ and p-tau levels as continuous variables in most of the statistical analyses. In addition, the cross-sectional design of the study did not permit an examination of changes in biomarker levels over time and their relation to clinical progression. Moreover, MMPs and TIMPs have previously been studied in both plasma and CSF; it would have strengthened our study to include paired CSF-plasma samples.

A major strength of the current study is the relatively large and well-characterised patient cohort, the age-matched CU control groups, and the use of a comprehensive standardised assessment for both patients and controls. There is good generalisability to other memory clinic populations. Further, follow-up data was available for most patients and the effects of baseline levels of the different MMPs and TIMPs on cognitive and functional decline could be determined. Finally, we have performed sex-stratified analyses in line with recommendations from the Women’s Brain Project [50, 51], highlighting potential sex-specific effects of certain MMPs and TIMPs in AD.

### Perspectives and significance

Our results support the use of MMPs/TIMPs as markers sex differences in AD pathology and possibly progression. Although numerous studies have explored MMPs in the CSF and in blood plasma as biomarkers of AD, our study highlight the importance of performing sex-stratified analysis to expand our understanding of the drivers of sex differences in AD pathology. Especially, our results suggest that sex-specific effects of these MMP-3 and TIMP-4 on amyloid pathology should be studied further. Moreover, our results demonstrate sex differences in the use of MMP-10 as a prognostic biomarker for AD, and we highlight that this marker should be further studied in relation to sex-specific disease mechanisms. Follow-up studies should explore the association between MMPs/TIMPs and known drivers of sex differences in AD such as sex hormones.

## Supplementary Information

**Additional file 1.** Supplementary information and tables.

## Data Availability

The data are not publicly available due to legal restrictions, imposed by the registry owners and the ethical committee, preventing us from publicly sharing the de-identified dataset due to sensitive patient information. The clinical data on the memory clinic patients may be requested from NorCog at e-mail: post@aldringoghelse.no. The demographic data for the CU controls and results of the MMP/TIMP analysis are available upon reasonable request to the authors. All data availability is dependent on the approval from the REC South East, contact at e-mail: post@helseforskning.etikkom.no.

## References

[1] Jack CR, Bennett DA, Blennow K (2018). NIA-AA research framework: toward a biological definition of Alzheimer’s disease. Alzheimers Dement.

[2] Rivera S, Khrestchatisky M, Kaczmarek L, Rosenberg GA, Jaworski DM (2010). Metzincin proteases and their inhibitors: foes or friends in nervous system physiology?. J Neurosci.

[3] Rivera S, García-González L, Khrestchatisky M, Baranger K (2019). Metalloproteinases and their tissue inhibitors in Alzheimer’s disease and other neurodegenerative disorders. Cell Mol Life Sci.

[4] Zipfel P, Rochais C, Baranger K, Rivera S, Dallemagne P (2020). Matrix metalloproteinases as new targets in Alzheimer’s disease: opportunities and challenges. J Med Chem.

[5] Agrawal SM, Lau L, Yong VW (2008). MMPs in the central nervous system: where the good guys go bad. Semin Cell Dev Biol.

[6] Dzwonek J, Rylski M, Kaczmarek L (2004). Matrix metalloproteinases and their endogenous inhibitors in neuronal physiology of the adult brain. FEBS Lett.

[7] Yong VW (2005). Metalloproteinases: mediators of pathology and regeneration in the CNS. Nat Rev Neurosci.

[8] Zhu B-L, Long Y, Luo W (2018). MMP13 inhibition rescues cognitive decline in Alzheimer transgenic mice via BACE1 regulation. Brain.

[9] Leake A, Morris CM, Whateley J (2000). Brain matrix metalloproteinase 1 levels are elevated in Alzheimer’s disease. Neurosci Lett.

[10] Yoshiyama Y, Asahina M, Hattori T (2000). Selective distribution of matrix metalloproteinase-3 (MMP-3) in Alzheimer’s disease brain. Acta Neuropathol.

[11] Yan P, Hu X, Song H (2006). Matrix metalloproteinase-9 degrades amyloid-β fibrils in vitro and compact plaques in situ. J Biol Chem.

[12] Hernandez-Guillamon M, Mawhirt S, Blais S (2015). Sequential amyloid-β degradation by the matrix metalloproteases MMP-2 and MMP-9. J Biol Chem.

[13] Helbecque N, Hermant X, Cottel D, Amouyel P (2003). The role of matrix metalloproteinase-9 in dementia. Neurosci Lett.

[14] Backstrom JR, Lim GP, Cullen MJ, Tökés ZA (1996). Matrix metalloproteinase-9 (MMP-9) is synthesized in neurons of the human hippocampus and is capable of degrading the amyloid-beta peptide (1–40). J Neurosci.

[15] Nübling G, Levin J, Bader B (2012). Limited cleavage of tau with matrix-metalloproteinase MMP-9, but not MMP-3, enhances tau oligomer formation. Exp Neurol.

[16] Terni B, Ferrer I (2015). Abnormal expression and distribution of MMP2 at initial stages of Alzheimer’s disease-related pathology. J Alzheimers Dis.

[17] Asahina M, Yoshiyama Y, Hattori T (2001). Expression of matrix metalloproteinase-9 and urinary-type plasminogen activator in Alzheimer’s disease brain. Clin Neuropathol.

[18] Peress N, Perillo E, Zucker S (1995). Localization of tissue inhibitor of matrix metalloproteinases in Alzheimer’s disease and normal brain. J Neuropathol Exp Neurol.

[19] Dunckley T, Beach TG, Ramsey KE (2006). Gene expression correlates of neurofibrillary tangles in Alzheimer’s disease. Neurobiol Aging.

[20] Hoe HS, Cooper MJ, Burns MP (2007). The metalloprotease inhibitor TIMP-3 regulates amyloid precursor protein and apolipoprotein E receptor proteolysis. J Neurosci.

[21] Py NA, Bonnet AE, Bernard A, et al. Differential spatio-temporal regulation of MMPs in the 5xFAD mouse model of Alzheimer’s disease: evidence for a pro-amyloidogenic role of MT1-MMP. Original Research. Front Aging Neurosci. 2014;6. 10.3389/fnagi.2014.00247 PMC4166961 25278878

[22] Mroczko B, Groblewska M, Zboch M (2014). Concentrations of matrix metalloproteinases and their tissue inhibitors in the cerebrospinal fluid of patients with Alzheimer’s disease. J Alzheimers Dis.

[23] Horstmann S, Budig L, Gardner H (2010). Matrix metalloproteinases in peripheral blood and cerebrospinal fluid in patients with Alzheimer’s disease. Int Psychogeriatr.

[24] Duits FH, Hernandez-Guillamon M, Montaner J (2015). Matrix metalloproteinases in Alzheimer’s disease and concurrent cerebral microbleeds. J Alzheimers Dis.

[25] Whelan CD, Mattsson N, Nagle MW (2019). Multiplex proteomics identifies novel CSF and plasma biomarkers of early Alzheimer’s disease. Acta Neuropathol Commun.

[26] Boström G, Freyhult E, Virhammar J (2021). Different inflammatory signatures in Alzheimer’s disease and frontotemporal dementia cerebrospinal fluid. J Alzheimer’s Dis JAD.

[27] Bjerke M, Zetterberg H, Edman Å, Blennow K, Wallin A, Andreasson U (2011). Cerebrospinal fluid matrix metalloproteinases and tissue inhibitor of metalloproteinases in combination with subcortical and cortical biomarkers in vascular dementia and Alzheimer’s disease. J Alzheimers Dis.

[28] Hanzel CE, Iulita MF, Eyjolfsdottir H (2014). Analysis of matrix metallo-proteases and the plasminogen system in mild cognitive impairment and Alzheimer’s disease cerebrospinal fluid. J Alzheimers Dis.

[29] Stomrud E, Björkqvist M, Janciauskiene S, Minthon L, Hansson O (2010). Alterations of matrix metalloproteinases in the healthy elderly with increased risk of prodromal Alzheimer’s disease. Alzheimers Res Ther.

[30] Lorenzl S, Albers DS, LeWitt PA (2003). Tissue inhibitors of matrix metalloproteinases are elevated in cerebrospinal fluid of neurodegenerative diseases. J Neurol Sci.

[31] Martino Adami PV, Orellana A, García P (2022). Matrix metalloproteinase 10 is linked to the risk of progression to dementia of the Alzheimer’s type. Brain.

[32] Mattsson N, Insel P, Nosheny R (2014). Effects of cerebrospinal fluid proteins on brain atrophy rates in cognitively healthy older adults. Neurobiol Aging.

[33] Trentini A, Manfrinato MC, Castellazzi M, Bellini T (2022). Sex-related differences of matrix metalloproteinases (MMPs): new perspectives for these biomarkers in cardiovascular and neurological diseases. J Pers Med..

[34] Rosenberg GA (2009). Matrix metalloproteinases and their multiple roles in neurodegenerative diseases. Lancet Neurol.

[35] Iulita MF, Ganesh A, Pentz R (2019). Identification and preliminary validation of a plasma profile associated with cognitive decline in dementia and at-risk individuals: a retrospective cohort analysis. J Alzheimers Dis.

[36] Pentz R, Iulita MF, Mikutra-Cencora M, Ducatenzeiler A, Bennett DA, Cuello AC (2021). A new role for matrix metalloproteinase-3 in the NGF metabolic pathway: proteolysis of mature NGF and sex-specific differences in the continuum of Alzheimer’s pathology. Neurobiol Dis.

[37] McKhann GM, Knopman DS, Chertkow H (2011). The diagnosis of dementia due to Alzheimer’s disease: recommendations from the National Institute on Aging-Alzheimer’s Association workgroups on diagnostic guidelines for Alzheimer’s disease. Alzheimers Dement.

[38] Albert MS, DeKosky ST, Dickson D (2011). The diagnosis of mild cognitive impairment due to Alzheimer’s disease: Recommendations from the National Institute on Aging-Alzheimer’s Association workgroups on diagnostic guidelines for Alzheimer’s disease. Alzheimers Dement.

[39] Medbøen IT, Persson K, Nåvik M (2022). Cohort profile: the Norwegian Registry of Persons Assessed for Cognitive Symptoms (NorCog)—a national research and quality registry with a biomaterial collection. BMJ Open.

[40] Idland AV, Sala-Llonch R, Borza T (2017). CSF neurofilament light levels predict hippocampal atrophy in cognitively healthy older adults. Neurobiol Aging.

[41] Mattsson N, Andreasson U, Persson S (2011). The Alzheimer’s Association external quality control program for cerebrospinal fluid biomarkers. Alzheimers Dement.

[42] Morris JC (1993). The Clinical Dementia Rating (CDR): current version and scoring rules. Neurology.

[43] Cedarbaum JM, Jaros M, Hernandez C (2013). Rationale for use of the Clinical Dementia Rating Sum of Boxes as a primary outcome measure for Alzheimer’s disease clinical trials. Alzheimers Dement.

[44] Mlekusch R, Humpel C (2009). Matrix metalloproteinases-2 and -3 are reduced in cerebrospinal fluid with low beta-amyloid1–42 levels. Neurosci Lett.

[45] Qin W, Jia X, Wang F (2015). Elevated plasma angiogenesis factors in Alzheimer’s disease. J Alzheimer’s Dis.

[46] Payami H, Montee KR, Kaye JA (1994). Alzheimer’s disease, apolipoprotein E4, and gender. JAMA.

[47] Farrer LA, Cupples LA, Haines JL (1997). Effects of age, sex, and ethnicity on the association between apolipoprotein E genotype and Alzheimer disease: a meta-analysis. JAMA.

[48] Riedel BC, Thompson PM, Brinton RD (2016). Age, APOE and sex: triad of risk of Alzheimer’s disease. J Steroid Biochem Mol Biol.

[49] Santaella A, Kuiperij HB, van Rumund A (2020). Inflammation biomarker discovery in Parkinson’s disease and atypical parkinsonisms. BMC Neurol.

[50] Ferretti MT, Iulita MF, Cavedo E (2018). Sex differences in Alzheimer disease—the gateway to precision medicine. Nat Rev Neurol.

[51] Ferretti MT, Martinkova J, Biskup E (2020). Sex and gender differences in Alzheimer’s disease: current challenges and implications for clinical practice: Position paper of the Dementia and Cognitive Disorders Panel of the European Academy of Neurology. Eur J Neurol.

