# Supplementary material for: Sex-specific associations of matrix metalloproteinases in Alzheimer’s disease
Source: Biol Sex Differ. 2023 May 23;14:35. doi: 10.1186/s13293-023-00514-x (PMC10207710; doi:10.1186/s13293-023-00514-x)
Supplement: Supplementary file 1 — Additional file 1. Supplementary information and tables. [file 13293_2023_514_MOESM1_ESM.docx]

Sex-specific associations of matrix metalloproteinases in Alzheimer’s disease
Supplementary information

Mari Aksnes,^1^ Trine H. Edwin,^2^ Ingvild Saltvedt,^3,4^ Rannveig S. Eldholm,^3,4^
Farrukh A. Chaudhry,^5^ Nathalie B. Halaas,^1, 2^ Marius Myrstad,^6, 7^ Leiv O. Watne^8^ and
Anne-Brita Knapskog^2^

**Author affiliations:**

1 Department of Geriatric Medicine, University of Oslo, 0315 Oslo, Norway

2 Department of Geriatric Medicine, Oslo University Hospital, 0450 Oslo, Norway

3 Department of Neuromedicine and Movement Science, Norwegian University of Science and Technology, 7030 Trondheim, Norway

4 Department of Geriatric Medicine, Clinic of Medicine, St. Olavs Hospital, Trondheim University Hospital, 7030 Trondheim, Norway

5 Department of Molecular Medicine, University of Oslo, 0315 Oslo, Norway

6 Department of Internal Medicine, Bærum Hospital, Vestre Viken Hospital Trust, 1346 Gjettum, Norway

7 Department of Medical Research, Bærum Hospital, Vestre Viken Hospital Trust, 1346 Gjettum, Norway

8 Institute of Clinical Medicine, Campus Ahus, University of Oslo, Oslo, Norway

9 Department of Geriatric Medicine, Akershus University Hospital, Lørenskog, Norway

Correspondence to: Mari Aksnes

Full address: P.O. Box 4956 Nydalen, OUS HF Ullevål sykehus, 0424 Oslo, Norway

E-mail [mari.aksnes@medisin.uio.no](mailto:mari.aksnes@medisin.uio.no)

**Supplementary Table 1. Analytical details for the quantification of MMPs and TIMPs***

|  | **LLOQ** | **ULOQ** | **Intra-assay CV** | **Inter-assay CV** |
| --- | --- | --- | --- | --- |
| **MMP-2** | 30.6 | 79747.4 | 3.7 | ** |
| **MMP-3** | 23.8 | 56800.0 | 8.7 | ** |
| **MMP-10** | 0.2 | 31600.0 | 19.7 | ** |
| **MMP-12** | 0.4 | 6575.0 | 57.6 | ** |
| **TIMP-1** | 4.87 | 119072.6 | 6.4 | ** |
| **TIMP-2** | 15.3 | 102429.0 | 4.7 | ** |
| **TIMP-3** | 44.4 | 31777.6 | 5.4 | ** |
| **TIMP-4** | 2.4 | 5300 | 3.2 | ** |

*Data is not reported for MMP-1, MMP-7, MMP-8, MMP-9 and MMP-13 as these markers were detectable in less than 40 % of samples and excluded from further analysis. **Eve Technologies do not report the inter-assay variability for each assay, but perform quality controls to assure that inter-assay variability falls within the range of 5-20 %. **Abbreviations:** CV: coefficient of variance; LLOQ: lower limit of quantification; MMP: matrix metalloproteinase; TIMP: tissue inhibitor of matrix metalloproteinase, ULOQ: upper limit of quantification.

**Supplementary Table 2. Clinical profile of the included men and women**

|  | **Women** | **Men** | **χ^2^*/*t (df)** | ***P* (Cohen’s *d*)** |
| --- | --- | --- | --- | --- |
| **N** | 194 | 162 |  |  |
| **Age** | 70.5 (6.2) | 71.3 (6.4) | -1.23 | 0.22 |
| ***APOE* ε4 n (%)**^a^ | 126 (71.6) | 89 (61.38) | 3.75 | 0.05 |
| **Education** | 12.4 (3.3) | 13.5 (4.2) | -2.55 | **0.01 (0.3)** |
| **MMSE** | 24.6 (4.4) | 24.9 (5.0) | -0.61 | 0.54 |
| **CDT** | 3.8 (1.4) | 3.9 (1.4) | -1.00 | 0.32 |
| **CDR-SB^b^** | 3.7 (1.8) | 4.2 (2.5) | -1.63 | 0.10 |
| **Stage** |  |  | 4.41 | 0.11 |
| **CU n (%)** | 46 (23.7) | 54 (33.3) |  |  |
| **MCI n (%)** | 27 (13.9) | 23 (14.2) |  |  |
| **Dementia n (%)** | 121 (62.4) | 85 (52.5) |  |  |
| **A+** | 163 (84.0) | 119 (73.5) | 5.98 | **0.01** |
| **T+** | 113 (58.3) | 90 (55.6) | 0.26 | 0.61 |
| **N+** | 134 (69.1) | 102 (63.0) | 1.47 | 0.23 |

Data reported is mean (standard deviation) unless otherwise indicated. *P-*values are for comparisons between women and men using χ^2^ (categorical variables) or t-tests (continuous variables). Significant differences in **bold.**

^a^*n* = 321, 35 missing genotype; ^b^*n =* 249, CDR-SB only available for memory clinic patients. **Abbreviations:** A+: biomarkers positive for Aβ pathology; Aβ: amyloid-β; AD: Alzheimer’s disease; *APOE*: apolipoprotein E; CDR-SB: clinical dementia rating scale sum of boxes; CDT; clock drawing test; CSF: cerebrospinal fluid; CU: cognitively unimpaired; MCI: mild cognitive impairment; MMSE: mini-mental status examination; N+: biomarkers positive for neurodegeneration; p-tau: phosphorylated tau; T+: biomarkers positive for tau pathology; t-tau: total tau.

**Supplementary Table 3. CSF MMP/TIMP levels in the included men and women**

|  | **Women** | **Men** | **t (df)** | ***P* (Cohen’s *d*)** |
| --- | --- | --- | --- | --- |
| **MMP-2 (ng/mL)** | 41.5 (8.5) | 48.5 (10.5) | -6.89 | **< 0.01 (0.75)** |
| **MMP-3 (pg/mL)** | 216.7 (127.6) | 266.9 (141.0) | -3.53 | **< 0.01 (0.38)** |
| **MMP-10 (pg/mL)** | 21.4 (15.7) | 27.9 (20.5) | -3.38 | **< 0.01 (0.36)** |
| **MMP-12 (pg/mL)** | 3.2 (4.2) | 2.7 (3.8) |  | 0.22 |
| **TIMP-1 (ng/mL)** | 55.8 (13.1) | 63.9 (14.0) | -5.59 | **< 0.01 (0.60)** |
| **TIMP-2 (ng/mL)** | 67.6 (8.8) | 74.2 (9.6) | -6.75 | **< 0.01 (0.72)** |
| **TIMP-3 (ng/mL)** | 16.0 (1.3) | 16.6 (1.2) | -5.31 | **< 0.01 (0.56)** |
| **TIMP-4 (ng/mL)** | 1.6 (0.4) | 1.6 (0.4) |  | 0.57 |

Data reported is mean (standard deviation). *P-*values are for comparisons between women and men using t-tests (continuous variables). Significant differences in **bold.**

**Abbreviations:** CSF: cerebrospinal fluid; MMP: matrix metalloproteinase; TIMP: tissue inhibitor of matrix metalloproteinase.
